# Relationship Between Internet Behaviors and Social Engagement in Middle-Aged and Older Adults in Taiwan

**DOI:** 10.3390/ijerph16030416

**Published:** 2019-01-31

**Authors:** Ching-Ju Chiu

**Affiliations:** Institute of Gerontology, College of Medicine, National Cheng Kung University, Tainan 70101, Taiwan; cjchiu@mail.ncku.edu.tw; Tel.: +886-6-2353535 (ext. 5739)

**Keywords:** health behaviors, information technology, international, social engagement, technology

## Abstract

*Aim:* To examine older adults’ Internet use patterns and its relationship with social engagement. *Methods:* Telephonic interview data of older Internet users from two urban and two rural areas were analyzed (*N* = 248). Cluster analysis was used to identify their Internet use patterns. Multinomial logistic regression identified characteristics associated with the Internet usage groups, and the multiple regression was used to examine if the Internet usage pattern was associated with social engagement in real life. *Results:* The majority of older adults in Taiwan using the Internet were considered Leisure users (32%). Others were Sporadic (26%), Instrumental (21%), and Eager users (21%). Leisure and Eager users, but not Instrumental users, had significantly higher scores on social engagement compared with Sporadic users after controlling for sociodemographic and behavioral covariates. Eager Internet users were associated with 22.8% increase in the social engagement level, and Leisure users were associated with 31.2% increase in the social engagement level. *Conclusions:* Older adults with different Internet behaviors were associated with distinct sociodemographic and social engagement behaviors. Causal relationship is warranted for further investigation.

## 1. Introduction

The past decades have witnessed a dramatic increase in the number of older adults learning and using new technologies [1] and consider that advanced technologies can enhance their quality of life [2]. There is an increasing trend of using the Internet to promote the health of older adults [3,4]. Existing studies also indicate perspectives from older adults regarding their eagerness to catch up with the new technology [5].

Nevertheless, an insight into older adults’ navigation through websites may provide valuable input for better technology-assisted behavioral interventions or health delivery for this population [6]. When technologies expand and converge into each other and more types of people use them for a variety of different things, it becomes harder to understand and assess the increasingly fragmented behavior. Applying a user-typology approach will produce a better understanding of the user [7]. To empirically distinguish and measure various types of Internet use enables a more precise and nuanced approach to Internet behavior [7]. Some of the existing studies have used diverse theoretical frameworks, such as diffusion of innovation theory, or the uses and gratifications theory [8,9] to characterize and explain the adoption and maintenance of Information and Communication Technology (ICT) used among participants of different sociodemographic and behavioral characteristics. Others have explored and discriminated user categories of Internet use according to users’ behaviors of surfing for Internet services using the technology acceptance model [10,11]. These studies utilized the questionnaire survey (with paper, online, email, or telephone), in-depth and in-depth follow-up interviews, or qualitative in-depth analysis, and generally concluded distinct Internet usage patterns were associated with different demographic groups [7,12].

Although evidence reported a common structure underlies user behavior among different countries, generally from the Western region [10,12], there was only one research study conducted in Asia, which took place in Singapore [13]. Moreover, the majority of their participants were children, adolescents, and young adults, with few studies from older populations [13,14,15]. The question remains as to whether the behavioral typology of the Internet usage of older adults in the Asian population may be different from the Western population.

In addition, how the behavioral typology of Internet usage is associated with social engagement has fewer research studies. Social engagement is an important factor of older adults’ health [16]. Among the handful of existing studies that examined Internet behaviors and social life in heterogeneous age groups, some have argued Internet use may erode involvement in face-to-face public life [17], but some have indicated that Internet behaviors may increase real-life social engagement, communication, and well-being [18,19] and may actually foster social participation. For older adults, a recent study using a randomized controlled trial indicates that ICT can enhance social connectivity and reduce loneliness [20]. Mechanisms suggested by a review study indicate that ICT reduces older adults’ social isolation by helping older adults connect to the outside world, gain social support, engage in activities of interest, and boost self-confidence [21].

However, culture influences the lifestyle, and the lifestyle influences a way to communicate and interact with technologies, and, thus, older adults in a different cultural context may have distinct patterns. Taiwan’s population is aging rapidly, and elderly adults also have become the fastest growing population using the Internet. Currently, there were more than 70% of middle-aged adults and more than 30% of the elderly using the Internet [22]. The Internet user class in middle-aged and older adults not only reflect a user typology, but also a lifestyle typology for understanding current and future generation of older adults when linking social behaviors. The purpose of this study was to fill the gap in the literature with an aim to answer the following questions. (1) What are the different user types among adults aged 50 and older in Taiwan concerning Internet usage? (2) How are the different user types associated with sociodemographic variables and Internet perspectives/experience? (3) How Internet use typology associated with daily social engagement? Findings from this study will provide an important reference for understanding technology use and behaviors among middle-aged and older adults in Asian countries.

## 2. Materials and Methods

### 2.1. Participants

**Figure 1:**
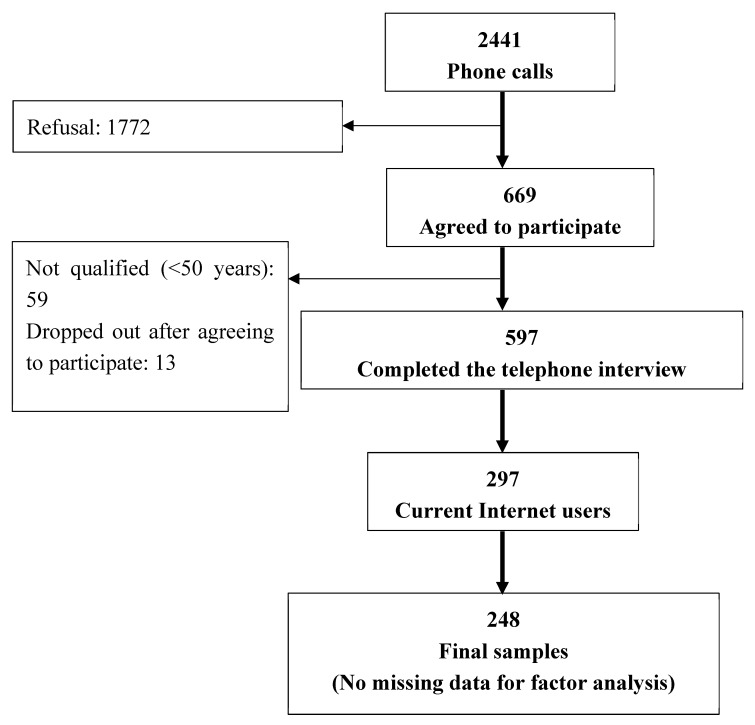
Flow diagram of the telephone interview and final analysis sample.

This study aims to interview participants who are representative of adults aged 50 and older in Taiwan who are using the Internet. To reach the participants as far as possible, the telephone interview was used. A telephone interview has the advantages of wide geographical access and reach hard to reach populations, such as shift workers or adults with difficulty to work with a face-to-face interview. The computer assisted telephone interview (CATI) procedure was used to enhance reporting and data quality. Phone directory listings was used to invite community-dwelling individuals from four randomly selected areas including two from the urban regions (i.e., Taipei and Tainan) and two from the suburban regions (i.e., Changhua and Taitung). From July to December 2014, a total of 2441 subjects were randomly invited from the phone book during this time period. A total of 597 (24.46%, 597/2441) participants aged 50 years or older agreed to complete the telephone survey, and a total of 297 (49.75%, 297/597) respondents were found to be current Internet users. Among them, data of the 248 (41.54%, 248/597) current Internet users with complete data were analyzed in this study. Figure 1 illustrates the detailed recruitment procedure and final sample. The proportion of these subjects’ experience in Internet use conformed to the Individual/Household Digital Opportunity Survey by the Research, Development, and Evaluation Commission, Taiwan [23]. This research was approved by the National Cheng Kung Hospital Institutional Review Board (No. B-ER-102-281).

### 2.2. Measures

To understand the Internet usage behaviors, the participants were asked, “Do you or have you participated in or used the following items?” Ten questions regarding their Internet use experience were presented and the participants were asked to rate each answer on a four-point scale, from 1 = very infrequently to 4 = very frequently. The 10 questions were further extracted for two factors (cumulative variance = 42.62%), which were renamed “leisure and recreation” (LR) and “information seeking” (IS). The LR variable was the mean score of five items (communication tool and apps, online learning, e-commerce for shopping, social network sites, and online video: α = 0.66). The IS variable was the mean score of five items (financial and economic, news, sanitation, nutrition, and foodstuffs, social welfare and retirement, and specific doctors’ reviews: α = 0.61). LR and IS were both measured using a four-point scale, which ranged from 1 = never, 2 = sometimes, 3 = often, and 4 = almost every day. Higher scores indicate greater leisure/recreation or information-seeking behavior.

The participants were also asked six questions regarding their perspectives on the benefits of the Internet in daily life using a five-point scale. The six questions extracted two factors (cumulative variance = 55.42%), which were “perspectives on increased social interaction” (it is convenient, to keep in touch with family more often, and to expand friendships: α = 0.53) and “perspectives on increased happiness” (it is pleasant, health status is better, and more plentiful leisure and recreation: α = 0.54). In addition, perspective on usefulness, suggested from the technology acceptance model [24] using a four-point Likert scale was also included.

Three more questions were used to measure participants’ Internet ability and experience. “Years of Internet use” was categorized as 0 = less than one year, 1 = one to two years, and 2 = three or more years. “Internet assistance needed” was categorized as 0 = not needed, 1 = needed, and 2 = completely needed. “Internet experience of relatives and friends,” was categorized as 0 = no, 1 = either relatives or friends, and 2 = both.

To measure the participants’ status of social engagement, the study adopted a social engagement questionnaire based on data obtained from the Survey of Health and Living Status of the Elderly in Taiwan developed by the Health Promotion Administration, Ministry of Health and Welfare, Taiwan, which has good validity and has been widely used [25,26]. The survey questionnaire included five items to measure participants’ social engagement, including participation in social club, religion activity, voluntary group, life-long learning, and exercise club. Each answer was scored using a five-point scale. Sociodemographic variables included age, gender, education level (Grades 1–6, Grades 7–9, Grades 10–12, and Grade 13 or above), living area (north, central, south, and east districts), cohabitation (solitary, with spouse only, and with children or spouse), and retirement (yes/no). A single-item scale with a five-point rating ranging from “strongly unsatisfactory” to “strongly satisfactory” was used to assess economic satisfaction.

### 2.3. Statistical Analyses

K-means clustering analysis was used to classify the participants’ behavioral patterns of surfing the Internet. Chi-square tests and ANOVA were used to describe characteristics associated with different Internet use types. A multinomial logistic regression analysis was conducted to determine the independent effect of each correlation in predicting the behavioral categories of the Internet usage types. Lastly, to examine the net effect of Internet user types in predicting daily social engagement, multiple regression analysis was used with social engagement as the dependent variable and factors that were associated with Internet user types as the covariates. Statistical analyses were performed using SAS version 9.4 (SAS Institute, Cary, NC, USA).

## 3. Results

The sample of 248 participants was 41.1% males. The average age was 62.73 (standard deviation (SD) = 9.40). At least 45.5% had some college education, completed college, or had graduate degrees. Most of the participants were with their children and spouse (65.7%) and retired (57.7%). The average for LR was 2.23 (SD = 0.64) and for IS it was 2.19 (SD = 0.66), which suggests that the participants’ Internet usage experience ranged from sometimes to often. Most of the participants’ Internet use history was ≥3 years (89.1%) and were not in need of Internet assistance (63.2%). The participants’ perspectives on happiness (mean: 3.40), usefulness (mean: 3.55), and increased social interaction (mean: 3.73) revealed that the Internet was more beneficial in their daily lives. The average for social engagement was 8.31 (total score = 25), which suggests that the participants had a low level of social engagement in their daily lives (see Table 1).

### 3.1. Behavioral Typology of Internet Usage

Based on the K-means cluster analysis with the LR and IS scores, four clusters of behaviors in surfing the Internet were identified: (1) Eager Users (20.97 %). In general, the mean scores of this user category were the highest for almost all Internet use behaviors. (2) Instrumental Users (20.97%): The IS mean score of this cluster was higher than the mean score of all the samples, such as financial and economic, news, and sanitation, nutrition, and foodstuffs, while the LR mean score was lower than the mean score of all the samples. (3) Leisure Users (32.26%): These users had a higher mean score in LR than the mean score of all the samples, such as participating in online courses and using Internet services (e.g., e-commerce, social networking sites, and communication software), while the IS mean score was lower than the mean score of all the samples. (4) Sporadic Users (25.81%): These participants had the lowest mean scores in IS and LR and they were characterized by their occasional and infrequent behaviors.

### 3.2. Characteristics Associated with Behavioral Typology of Internet Usage

As Table 2 shows, although the participants in the four categories were not different in their living areas, cohabitation status, and their perspectives on whether the Internet increased happiness, males tended to be Instrumental Users (50%). Females were more likely to be Leisure Users (71.3%). Participants with a lower education level were more likely to be Sporadic Users (9.4%) while participants with a higher education level were more likely to be Eager (65.38%) and Leisure Users (55.7%). Most of the retired participants were Eager (69.2%) and Instrumental users (73.1%) and most of those not retired were Leisure users (53.8%). Among those with higher scores of economic satisfaction, they were more likely to be Leisure (3.27%) and Eager users (3.26%). In addition, the participants whose Internet use was ≥3 years were more likely to be Eager (98.1%) and Leisure users (95%) and they did not need Internet assistance, while the participants who were completely in need of Internet assistance were more likely to be Sporadic (7.8%) and Instrumental users (7.7%) compared with Eager (0%) and Leisure users (1.3%). Moreover, the participants who had more relatives or friends surfing the Internet were more likely to be Eager (100%), Leisure (89.9%), and Instrumental Users (86.5%). The participants who reported the Internet was more useful and who had perspectives of increased social interaction were more likely to be Eager (3.79% and 3.95%, respectively) and Leisure users (3.73% and 3.83%, respectively). The participants who had increased their actual social engagement were more likely to be Eager (8.65%) and Leisure users (8.93%).

### 3.3. Factors Predicting Behavioral Typology of Internet Usage

As Table 3 presents, participants with a higher education level were significantly more likely to be Leisure users. Those who used the Internet ≥3 years were significantly more likely to be Leisure users. Participants who had more positive perspectives on the Internet being useful were significantly more likely to be Eager users while those who had a positive perspective on whether the Internet increased social interaction were significantly more likely to be Eager and Leisure users.

### 3.4. Can Internet Usage Typology Predict Daily Social Engagement?

As Table 4 shows, the behavioral typology of Internet usage was included in the multiple regression analysis to realize its independent specific effects on predicting social engagement in daily life, considering sociodemographic and Internet experience and perspective variables. Over and above the effect of sociodemographic factors and Internet experience, Internet usage typology was a significant factor in predicting daily social engagement. Eager and Leisure users had 1.14 (*p* = 0.040) and 1.56 (*p* = 0.002) higher social engagement scores, respectively, compared with Sporadic users.

## 4. Discussion

There is a void in the literature providing population-based evidence on the relationship between Internet usage type and social engagement in middle-aged and older adults. This study assesses Internet use over a wide range of participants in Taiwan, and examines the associations of Internet usage typology with social engagement behaviors net of the effects of a wide range of potential confounders. Several key results have emerged from this investigation.

Among middle-aged and older adults using the Internet in Taiwan, four different categories were identified in light of their various patterns of Internet usage: Leisure users (32%, they participated in online courses and used e-commerce, social networking sites, and communication software), Sporadic users (26%, they had occasional and infrequent Internet use behaviors), Instrumental users (21%, they used the Internet to search for financial and economic items, news, and sanitation, nutrition, and foodstuffs), and Eager users (21%, they engaged in almost all of the Internet use behaviors). Comparison between results from the present study and that from previous studies in Europe (Norway, Sweden, Austria, the UK, and Spain) [10], it is interesting to find that the Internet use category of Leisure was the highest in Taiwan (32.26%), while this category was the lowest in Europe (17%). Instrumental users was the lowest in Taiwan (20.97%), while it was the highest in Europe (31%) and Sporadic users was the second most frequent in Taiwan (25.81%) despite being the highest in Europe (31%).

This study found that Instrumental Users consisted of mostly male and retired elders. Leisure users consisted mostly of female and non-retired elders. Eager/Leisure users had higher education levels, economic satisfaction, more years of Internet use, no need for Internet assistance, perceived more usefulness/increased social interaction, and were more likely to increase their social engagement in actual social life. Eager/Leisure/Instrumental users had more relatives and friends who also surfed the Internet. Different from previous studies, in Europe, more females than males tended to be Instrumental users [10]. The current study found that older males tended to be Instrumental users (50%) and older females were significantly more likely to be Leisure users (71.3%). This finding can be explained by the diversity of the nation and the age groups. Education seemed to be a crucial factor for ICT adoption by older adults, and, as such, this should be taken into account in any initiative to bridge the digital divide [27]. Previous studies in the Netherlands and Sweden both found that older adults/patients with a lower level of education were less likely to use e-Health, while those with a higher level of education tended to use the Internet more often compared with middle-educated or low-educated older adults [28,29,30]. The findings from the current study echo previous studies, which indicates that middle-aged and older adults in Taiwan with a lower education level most often tended to be Sporadic users, while, with a higher education level, participants most often tended to be Eager and Leisure users.

Importantly, this study supported the hypothesis that active use of the Internet may be associated with increased social engagement in daily life. Eager users and Leisure users had 1.14 and 1.56 significantly higher scores, respectively, on social engagement in their daily lives. In other words, eager Internet users were associated with 22.8% (1.14/5) increase in the social engagement level, and leisure users were associated with 31.2% (1.56/5) increase in the social engagement level. This association remained significant after controlling for a wide range of potential demographic and behavioral factors and is consistent with previous studies that showed a positive association between Internet use and frequency of contact with others [7,26,27], which suggests the importance of promoting more Internet use among older adults to increase active aging.

This is the first study to examine the behavioral typology of Internet use in older adults using the Internet in non-Western populations and, with nationwide sampling, it made it possible to assess Internet use over a wide range of participants in Taiwan. Policy decisions aim to reduce inequalities in access to and use of information technologies that must take into consideration the necessary investment in training and support as well. This research has underlined the importance of the older population segment and their different Internet usage characteristics and needs. For example, providing the technology industry and educators with information relating to different Internet usage typologies and sociodemographic profiles will encourage the development of genuinely usable ICT products and training and support approaches that could potentially help achieve active aging.

However, there were several limitations in the current study. First, this study utilized a self-report measure of the data (e.g., the social engagement data). Although self-report is a commonly used method for collecting behavioral and attitude-related data of participants living in the community, it should be noted that socially desirable responses may be incurred and may limit reliability [31,32,33,34,35]. Second, the study was based on snap-shot, cross-sectional research design and may not have drawn any conclusions on the causal relationship between Internet user types and social engagement. Third, although this study aims to collect participants who were representative of older adults living in different areas in Taiwan, due to the limitation of the telephone interview, adults who did not have a local phone were not explored. Similarly, older adults who agree to participate in research of this type may be a select group due to a relatively lower response rate compared with in-person interviews [36]. For example, participants in the present study were more educated than the general older adult population in Taiwan (45.5% had at least a college level education). Future researchers are encouraged to use alternative sampling methods and longitudinal datasets to reach underrepresented populations, and to confirm causal relationship. Last, the present study was limited by its cross-sectional data collection, and, thus, was not able to differentiate older adults’ Internet usage behaviors by their long-term change pattern. The older adults’ behavior is a changing process, and, thus, if longitudinal data is available, it is encouraged to identify longitudinal change patterns and to identify subgroups with the repeated measures of the same panel of participants and with more advanced statistics such as the latent class analysis (LCA). It is acknowledged that the cluster analysis employed in the present study has the same strength with the LCA to identify subgroups in the whole population but with the limitation to omit potential change related to time.

In conclusion, this research has underlined the importance of the middle-aged and older population segment regarding their different characteristics and needs using the Internet. In addition, Internet usage among adults aged 50 and over is associated with social engagement in daily life, which suggests the possibility of promoting more Internet use among middle-aged and older adults to increase active aging.

## Figures and Tables

**Table 1:** Participants’ sociodemographic characteristics, Internet experience and perspective, and social engagement.

Variables	% or Means (SD)
***Sociodemographic Factors***	
Age	62.73 (9.40)
*Gender*	
Male	41.1
Female	58.9
*Education level*	
1–6 years	4.5
7–9 years	11.0
10–12 years	39.0
13+ years	45.5
*Living area*	
North district	32.7
Central district	23.4
South district	25.8
Eastern district	18.1
*Cohabitation*	
Solitary	7.8
With spouse only	26.5
With children or spouse	65.7
*Retirement*	
Yes	57.7
No	42.3
Economic satisfaction (range: 1–5)	3.14 (0.70)
**Internet Experience and Perspectives**	
Leisure and recreation (LR, range: 1–4)	2.23 (0.64)
Information seeking (IS, range: 1–4)	2.19 (0.66)
*Internet use history*	
< 1 year	4.8
1~2 years	6.1
3+ years	89.1
*Internet assistance need*	
No need	63.2
Need	32.8
Completely need	4.1
*Internet experience of relatives and friends*	
No	1.2
Either relatives or friends	11.7
Both	87.0
Perspective on increased happiness (range: 1–5)	3.40 (0.57)
Perspective on usefulness (range: 1–4)	3.55 (0.65)
Perspective on increased social interaction (range: 1–5)	3.73 (0.58)
**Social Engagement** (range: 5–25)	8.31 (2.80)

SD—standard deviation. LR—leisure and recreation. IS—information seeking.

**Table 2:** Typology of Internet use among middle-aged and older adults and their association with sociodemographic, Internet experience/perspectives, and social engagement factors.

Variables	Means (SD)					Post Hoc
	Eager (n = 52, 21%)	Instrumental (n = 52, 21%)	Leisure (n = 80, 32%)	Sporadic (n = 64, 26%)		
**Sociodemographic Characteristics**						
Age (range: 50–105)	62.52 (11.82)	65.58 (10.34)	60.56 (6.47)	63.28 (9.03)	F = 3.17 *	2 > 3
*Gender*						
Male	22(42.31)	26(50.00)	23(28.75)	31(48.44)	χ^2^ = 8.195 *	
Female	30(57.69)	26(50.00)	57(71.25)	33(51.56)		
*Education level*						
1–6 years	2(3.85)	2(3.92)	1(1.27)	6(9.38)	χ^2^ = 35.380 ***	
7–9 years	0(0.0)	5(9.80)	8(10.13)	14(21.88)		
10–12 years	16(30.77)	26(51.98)	26(32.91)	28(43.75)		
13+ years	34(65.38)	18(35.29)	44(55.70)	16(25.00)		
*Living area*						
Taipei	23(44.23)	16(30.77)	26(32.50)	16(25.00)	χ^2^ = 11.349	
Changhua	13(25.00)	15(28.85)	14(17.50)	16(25.00)		
Tainan	11(21.15)	12(23.08)	20(25.00)	21(32.81)		
Taitung	5(9.62)	9(17.31)	20(25.00)	99(17.19)		
*Cohabitation*						
Solitary	6(11.76)	2(3.92)	6(7.59)	5(7.81)	χ^2^ = 8.217	
With spouse only	17(33.33)	18(35.29)	15(18.99)	15(23.44)		
With children or spouse	28(54.90)	31(60.78)	58(73.42)	44(68.75)		
*Retirement*						
Yes	36(69.23)	38(73.08)	37(46.25)	32(50.00)	χ^2^ = 13.719 **	
No	16(30.77)	14(26.92)	43(53.75)	32(50.00)		
Economic satisfaction score (range: 1–5)	3.26 (0.78)	3.08 (0.65)	3.27 (0.66)	2.95 (0.70)	F = 3.12 *	(1,3) > 4
**Internet Experience and Perspectives**						
*Internet use history*						
< 1 year	1(1.92)	5(3.85)	1(1.25)	8(12.50)	χ^2^ = 20.329 **	
1–2 years	0(0.0)	5(9.62)	3(3.75)	7(10.94)		
3+ years	51(98.08)	45(86.54)	76(95.00)	49(76.56)		
*Internet assistance need*						
No need	40(78.43)	27(51.92)	57(71.25)	32(50.00)	χ^2^ = 18.343 **	
Need	11(21.57)	21(40.38)	22(27.50)	27(42.19)		
Completely need	0(0.0)	4(7.69)	1(1.25)	5(7.81)		
*Internet experience of relatives and friends*						
No	0(0.0)	2(3.85)	0(0)	1(1.56)	χ^2^ = 23.122 ***	
Either relatives or friends	0(0.0)	5(9.62)	8(0.13)	16(25.00)		
Both	52(100.0)	45(86.54)	71(87)	47(73.44)		
Perspectives on increased happiness (range: 1–5)	3.50 (0.55)	3.37 (0.56)	3.45 (0.56)	3.29 (0.60)	F = 1.54	
Perspectives on usefulness (range: 1–4)	3.79 (0.41)	3.33 (0.71)	3.73 (0.50)	3.30 (0.77)	F = 10.55 ***	1 > (2,4) 3 > (2,4)
Perspectives on increased social interaction (range: 1–5)	3.95 (0.55)	3.56 (0.61)	3.83 (0.58)	3.56 (0.49)	F = 7.12 ***	1 > (2,4) 3 > (2,4)
**Social Engagement** (range: 5–25)	8.65 (2.67)	8.00 (2.57)	8.93 (2.99)	7.53 (2.69)	F = 3.51 *	(1,3) > 4

Note: Numbers are mean (SD) or count (percentage). F statistics by ANOVA; χ^2^—chi-square test statistics. * *p* < 0.05, ** *p* < 0.01, *** *p* < 0.001.

**Table 3:** Factors predicting participants in different Internet usage types.

	Eager/Sporadic		Instrumental/Sporadic		Leisure/Sporadic	
	OR (95% CI)		OR (95% CI)		OR (95% CI)	
*Gender*						
Male/Female	0.91	(0.36–2.30)	1.01	(0.44–2.32)	0.52	(0.23–1.20)
*Age*	0.98	(0.93–1.03)	1.00	(0.96–1.05)	0.97	(0.92–1.02)
*Education level*						
7–9 years/1–6 years	0.00	(0.00–0.00)	0.89	(0.12–6.58)	5.12	(0.40–66.41)
10–12 years/1–6 years	1.16	(0.16–8.57)	3.19	(0.56–18.12)	4.90	(0.45–53.69)
13+ years/1–6 years	4.51	(0.58–35.16)	3.73	(0.59–23.45)	17.73 *	(1.54–203.50)
*Retirement*						
Yes/No	1.85	(0.68–4.99)	2.13	(0.83–5.45)	0.69	(0.28–1.69)
*Internet use history*						
1–2 years/<1 year	0.00	(0.00–0.00)	1.80	(0.21–15.48)	4.93	(0.31–78.25)
3+ years/<1 year	2.37	(0.19–29.48)	2.02	(0.33–12.29)	11.56 *	(1.08–123.30)
Perspectives on increased happiness	1.57	(0.68–3.65)	1.65	(0.79–3.44)	1.45	(0.70–2.99)
Perspectives on usefulness	2.66 *	(1.15–6.14)	0.85	(0.48–1.52)	1.77	(0.91–3.46)
Perspectives on increased social interaction	3.61 **	(1.52–8.56)	1.03	(0.49–2.14)	2.62 *	(1.23–5.57)

* *p* < 0.05, ** *p* < 0.01, *** *p* < 0.001. OR—Odds Ratio, CI—Confidence Interval.

**Table 4:** Net effect of Internet usage typology in predicting social engagement score.

	**β**	**SE**	**P value**
**Sociodemographic Characteristics**			
*Gender*			
Male/Female	−1.34 ***	0.36	0.0003
*Age*	0.03	0.02	0.2197
*Education level*			
7–9 years/1–6 years	−0.57	0.96	0.5494
10–12 years/1–6 years	−0.35	0.85	0.6864
13+ years/1–6 years	−0.69	0.88	0.4322
*Retirement*			
Yes/No	−1.44 ***	0.38	0.0002
**Internet Experience and Perspectives**			
*Internet use history*			
1–2 years/< 1 year	0.28	1.07	0.7969
3+ years/< 1 year	−0.73	0.85	0.3943
Perspectives on increased happiness	−0.03	0.32	0.9173
Perspectives on usefulness	0.16	0.29	0.5949
Perspectives on increased social interaction	−0.33	0.33	0.3200
**Behavioral Typology of Internet Usage**			
Eager/Sporadic	1.14 *	0.55	0.0404
Instrumental/Sporadic	0.28	0.51	0.5911
Leisure/Sporadic	1.56 **	0.49	0.0016

SE—Standard Error. β—regression coefficient. *p < 0.05. **p < 0.01. ***p < 0.001.
